# Supplementary material for: Deterministic and robust room-temperature exchange coupling in monodomain multiferroic BiFeO3 heterostructures
Source: Nat Commun. 2017 Nov 17;8:1583. doi: 10.1038/s41467-017-01581-6 (PMC5691063; doi:10.1038/s41467-017-01581-6)
Supplement: Supplementary file 1 — Supplementary Information [file 41467_2017_1581_MOESM1_ESM.pdf]

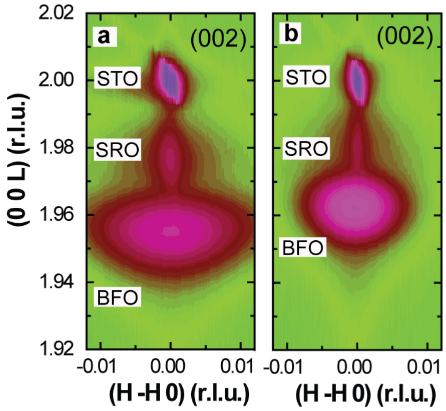

Supplementary Figure 1. Strain states for $BiFeO_3$ thin films. Reciprocal space mapping (RSM) by four-circle x-ray diffraction for (**a**) 165 nm and (**b**) 300 nm thick films. The critical thickness for fully–strained $BiFeO_3$ films on $SrTiO_3$ (001) is ~50 nm, with strain relaxing gradually until the thickness exceeds ~800 nm[1]. As discussed in Jang *et.al*, one mechanism for strain relaxation is a crystallographic tilt by dislocation multiplication along $[110]_{pc}$ (the miscut direction). The lattice parameters, α, β and tilted angles (δ) are shown in Supplementary Table 1. $BiFeO_3$ films 165 nm and 300 nm thick are partially coherent to the substrates; with increasing film thickness, the out-of-plane parameters decrease due to the relaxation of biaxial compressive strains.

Thickness (nm)	$a_{[100]}$ (Å)	$b_{[010]}$ (Å)	$c_{[001]}$ (Å)	α (°)	β (°)	$\delta_{[110]}$ (°)
165	3.944	3.945	3.990	89.43	89.44	0.11
300	3.959	3.955	3.982	89.39	89.41	0.13
Bulk	3.960	3.960	3.960	89.40	89.40	-

Supplementary Table 1. $BiFeO_3$ lattice parameters. Measured lattice parameters *a*, *b* and *c*, angles α, β and tilt angle δ of 165 nm, 300 nm and bulk $BiFeO_3$.

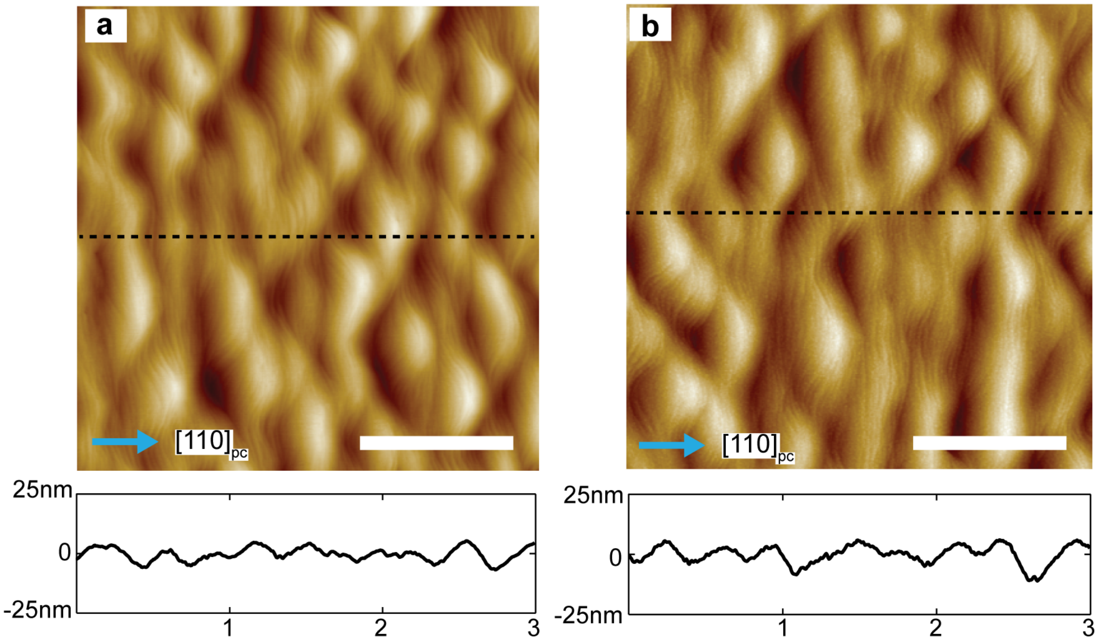

Supplementary Figure 2. Surface morphology of the heterostructures. Atomic force microscope (AFM) images showing smooth surface morphology of both (**a**) as-grown $BiFeO_3$ (RMS roughness ~ 4.0 nm) and (**b**) after Al (3nm)/Co (2nm) deposition on $BiFeO_3$ (RMS roughness ~4.3 nm). Scale bar, 1 μm. During $BiFeO_3$ growth on miscut $SrTiO_3$ substrates, step bunching occurs to accommodate structural relaxation[2]. Smooth surfaces both before and after Al/Co deposition on top of $BiFeO_3$ are seen.

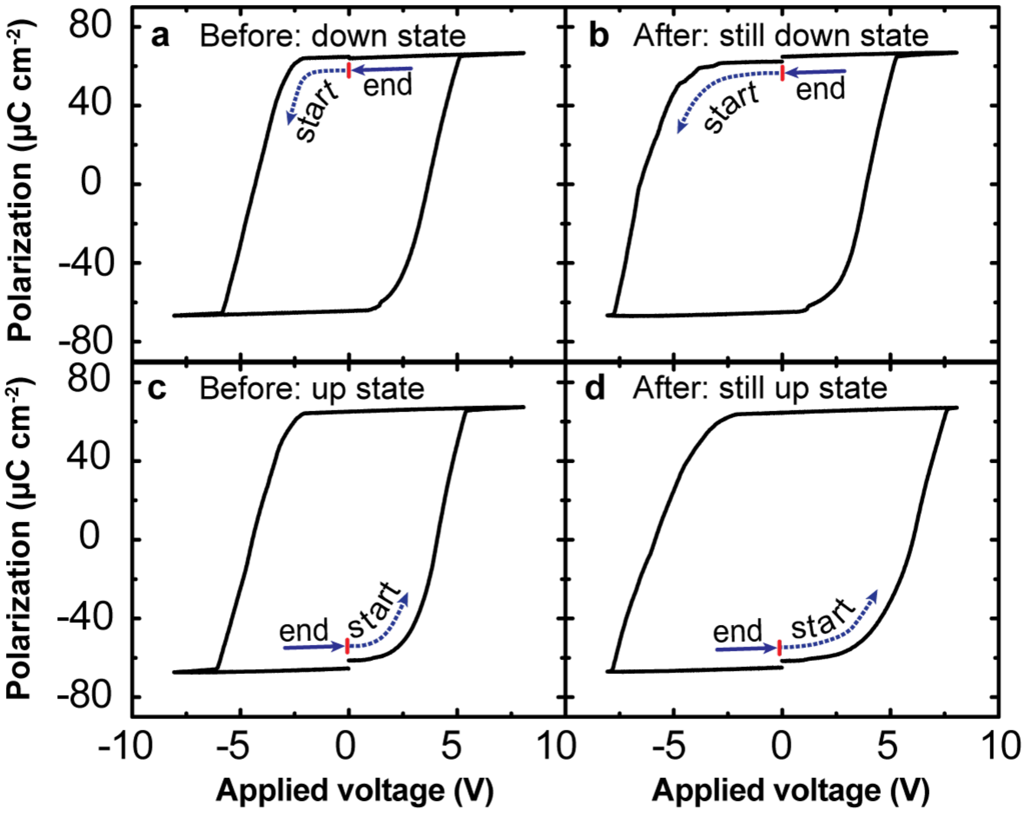

Supplementary Figure 3. Stable polarization states before and after PEEM measurements. A Ferroelectric Tester (Radiant Technologies, Inc., Albuquerque NM) was mounted in the high-voltage rack of an Elmitec LEEM-PEEM III to permit *in situ* measurement of polarization versus voltage loops on the patterned samples (total device area ~100x500 μm^2) before and after XMLD and XMCD PEEM imaging. The *in situ* polarization versus applied voltage hysteresis loops measurement are shown, (**a**) and (**c**) taken before the PEEM measurements to set the polarization to down and up states, respectively. (**b**) and (**d**) show the hysteresis loops after the measurements confirming that the polarization state is stable in the down state and up states, respectively. P–V loops taken with 1 ms period at 120 K; $BiFeO_3$ thickness is 300 nm, with 10 devices patterned on each 5x5 mm^2 substrate.

Supplementary Note 1. Polarization domain state.

To determine the ferroelectric domain distribution of $BiFeO_3$ film in the virgin, up and down states, piezoresponse force microscopy (PFM) was performed. The scan area of 12 μm x 12 μm was chosen to show the ferroelectric domains in the largest area that the microscope can provide, and allows confirmation that the $BiFeO_3$ film has a uniform domain at least over this large area. After polarization switching from down to up state, the out-of-plane PFM image (Supplementary Fig. 4b-c) shows only one contrast indicating a 100% switching out of the plane component along $[001]_{pc}$ direction. While the in-plane PFM image (Supplementary Fig. 4c) has two contrast levels; the majority darker contrast and the minority bright contrast. Combining information from Supplementary Figure 4b and 4c, the up polarization state has two domains with a majority (97%) r_3^+ domain and minority (3%) r_1^+ domain. After switching from up to down polarization state, both the out-of-plane and in-plane PFM images (Supplementary Fig. 4d) show only one contrast, and have the same contrast as the unswitched area. This confirms that the polarization of the switched area from the up to down state has the same polarization as the virgin state.

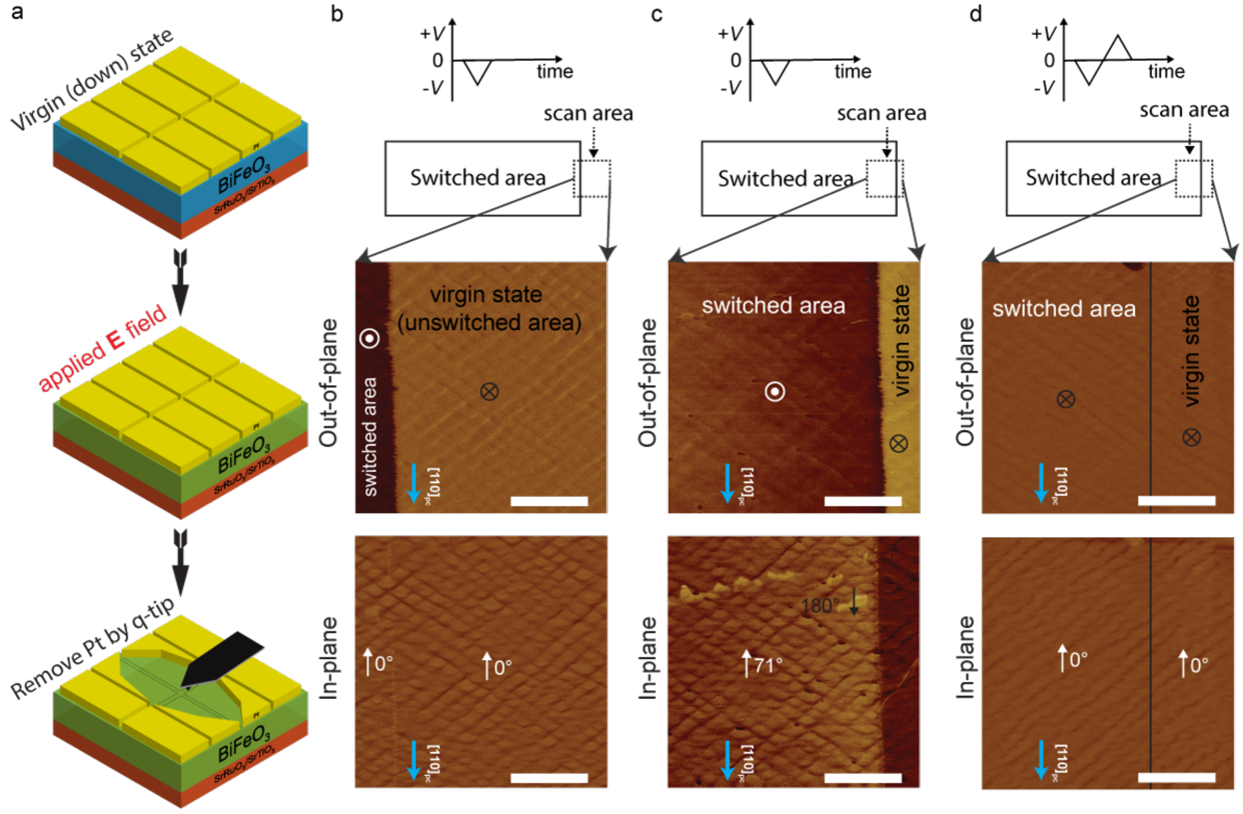

Supplementary Figure 4. Ferroelectric domain of "virgin"-"up"-"down" polarization states. (**a**) Schematic of sample preparation for piezoresponse force microscope (PFM) measurement performed at room temperature on $BiFeO_3$ (300 nm). A Pt top electrode is photolithographically patterned by lift-off. The electric field is applied via Pt top electrode for desired polarization as performed with triangular waveform. The Pt electrodes are then removed by rubbing with q-tip. (**b**) The schematic of scan area focusing on the unswitched area is shown. PFM images of out-of-plane and in-plane of the virgin (unswitched) state has one contrast, confirming that the $BiFeO_3$ film is ferroelectrically monodomain. The dark contrast (switched area) of the out-of-plane image is also shown, to facilitate the interpretation. (**c**) The schematic of scan area focusing on the switched area is shown. The out-of-plane PFM image shows single ferroelectric domain while the in-plane PFM image exhibits 97% ferroelectric (majority) –71° switching (darker contrast-white arrow) and 3% of minority domains –180° switching (bright contrast-black arrow). (**d**) The schematic of scan area focusing on the switched area is shown. After switching from up to down polarization state, both the out-of-plane and in-plane PFM images show only one contrast and has the same contrast as the unswitched area. This confirms

that the polarization of the switched area from up to down state has the same polarization as the virgin state. Note that PFM images in **b-c-d** are measured from the same sample but different electrodes. The dark contrast from the out-of-plane PFM image in **d** is the Pt electrode residue. Scale bars, 4 μm.

Supplementary Note 2. Bulk Cycloid orientation of $BiFeO_3$ determined using Neutron Diffraction.

The magnetic structure present in the bulk of the thin film was determined by time-of-flight single crystal neutron diffraction. This technique provides sensitivity to the full $BiFeO_3$ film volume, and is therefore complementary to the surface sensitive XMLD and XMCD PEEM measurements described below, enabling us to gain a full overview of the magnetic properties of the film. Two separated 300 nm $BiFeO_3$ films were studied, pre-poled the entire sample in the down and up states, respectively. In each case, the film was systematically aligned such that ±**k** magnetic diffraction satellites (**k** is the cycloidal propagation vector) of the four structurally-forbidden $(½,± ½,± ½)_{pc}$ reflections could be measured. In all cases, we only observed a single pair of magnetic satellites (see Fig. 2a and 2b), oriented parallel to $[1\text{-}10]_{pc}$ (i.e., perpendicular to the miscut direction); this indicates that each film supports a single cycloidal domain rather than the three domains observed in bulk single crystals[3].

The rhombohedral distortion of the pseudo-cubic cell, which corresponds to a single ferroelastic domain (or equivalently to two 180° ferroelectric domains), results in different *d*-spacings for the four $(½,± ½,± ½)_{pc}$ diffraction planes since the four reciprocal lattice vectors are no longer equivalent in the rhombohedral lattice. Therefore, the population of all possible ferroelastic domains could be directly determined by plotting the diffraction data as a function of *d*-spacing. The down state sample comprised a single ferroelastic domain with the polar axis parallel to the miscut direction (orthogonal to the substrate steps) when projected onto the surface of the film. By contrast, the up state sample comprised of a majority (88%) ferroelastic domain, with polar axis also parallel to the miscut direction when projected onto the film surface, but switched up 71° with respect to the down state. The other 12% of the sample either remained unswitched, or was switched by 180°.

The real space periodicity of the magnetic cycloids was calculated from the separation of the magnetic satellites in reciprocal space (Fig. 2a (bottom pane) and 2b (bottom pane)). In both

up and down states the cycloid was found to have the same periodicity of 656(8) Å (using a_{hex} = 5.5799(3) Å, Ref. 4), which is extended by 6% compared to the cycloidal periodicity observed in bulk single crystals (620 Å, Ref. 5).

Since neutron diffraction intensity is proportional to the square of the Fourier components of the magnetization orthogonal to the scattering vector, the relative intensities of the four sets of magnetic satellite reflections may be used to calculate the plane of rotation of the cycloid. Fig. 2c shows fits to the diffraction peaks measured from the down film. Here, the plane of the cycloid was found to be inclined from the plane of the film by +12(2)° towards the electric polarization vector. This is remarkably different from the bulk, where the plane of the cycloid contains the electric polarization and is therefore inclined by +36° using the same conventions. The most plausible interpretation of this discrepancy is the presence of an additional magnetic anisotropy that favors magnetic moments lying within the plane of the film. In the up film (Fig. 2d), the cycloidal plane was found to be essentially in the plane of the film (the refined inclination angle was +1(2)° with the same conventions).

To summarize, upon switching the electric polarization by 71° between two single, magneto-elastic domains, the magnetic cycloid in the bulk of the film rotates about its propagation vector following the direction of the electric polarization. However, the cycloidal plane is much closer to the plane of the film than in the bulk and the switching angle is much smaller (13° in the film vs 71° in the bulk), indicating the presence of a significant anisotropy in the plane of the film.

Supplementary Note 3. Magnetic anisotropy of Co on (001) $SrTiO_3$ 4° miscut toward $[110]_{pc}$ direction.

The full $BiFeO_3$ heterostuctures investigated in this work have a step morphology because they are grown on miscut substrates. When the $BiFeO_3$ is in the down state there is a magnetic anisotropy parallel to the steps. Even though magnetic anisotropy in ferromagnetic thin films grown on miscut substrates is a well-studied phenomena[6], it is prudent to test whether the anisotropy parallel to the steps is indeed caused by the steps especially since $BiFeO_3$ has magnetic order. This test was carried out by growing unpatterned Co films on an annealed (001) $SrTiO_3$ 4° miscut toward $[110]_{pc}$ direction substrates. The surface morphology of both before and after Al/Co deposited on an annealed (001) $SrTiO_3$ is shown in Supplementary Figure 5. The

annealed high miscut substrate shows a step bunching with step edges perpendicular to $[011]_{pc}$ miscut direction while after Al/Co deposition the surface is rougher with a resemblance of the morphology to that before the deposition.

MOKE **M-H** loops showed magnetic anisotropy parallel to the step edges. The coercive field of the loop measured with **H** parallel to step edges is 1.60 mT. This value is smaller when compares to the **M-H** loop of $Co/BiFeO_3$ heterostructure as shown in Supplementary Figure 6. The larger coercive field in the heterostructure is known as an exchange enhancement which is one of the evidence showing that Co and $BiFeO_3$ has the exchange interaction.

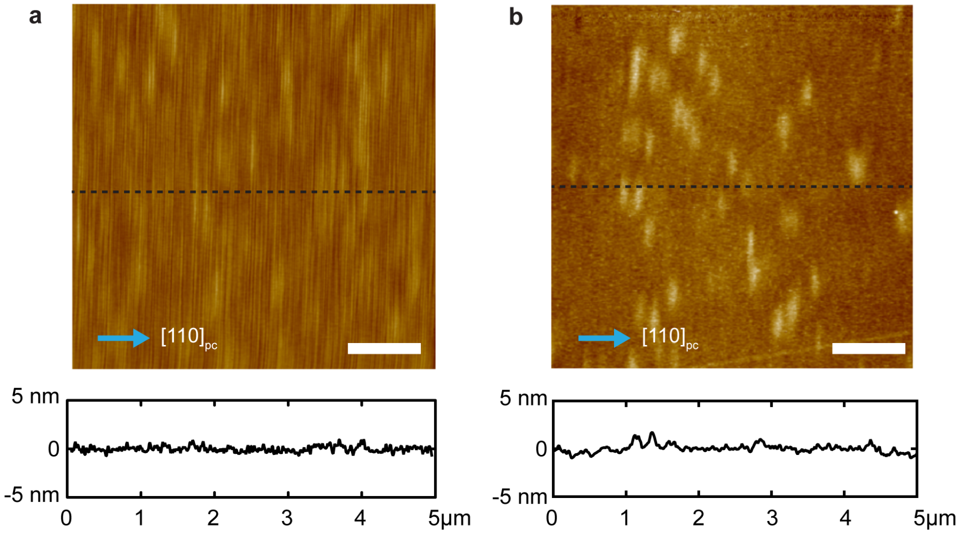

Supplementary Figure 5. Surface morphology of annealed high miscut $SrTiO_3$ substrate with and without Al/Co overlayer. Atomic force microscope (AFM) images showing surface morphology of both (**a**) annealed (001) $SrTiO_3$ 4° miscut toward $[110]_{pc}$ direction substrates (RMS roughness ~ 0.2 nm) and (**b**) after Al (3 nm)/Co (2 nm) deposition on top (RMS roughness ~0.5 nm). Scale bars, 1 μm.

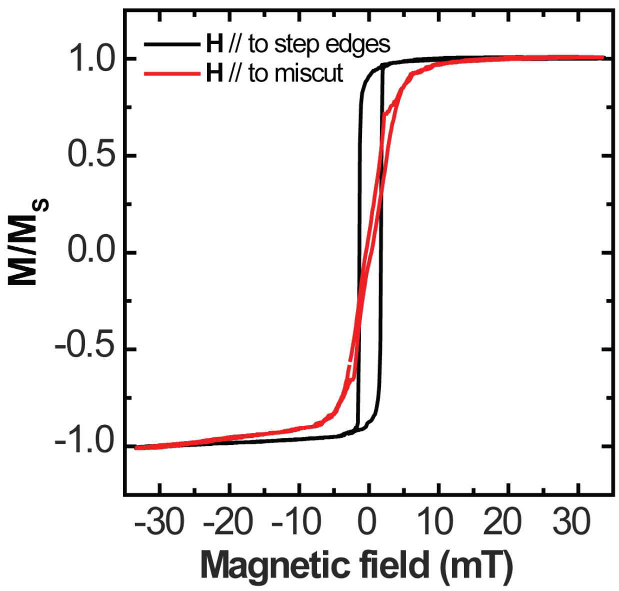

Supplementary Figure 6. MOKE of Co on miscut $SrTiO_3$. Experimental MOKE **M-H** loops of the Co (2 nm) deposited on top of (001) $SrTiO_3$ 4° miscut toward [110] measured with the applied magnetic field parallel to and perpendicular to the substrate step edges.

Supplementary Note 4. Interpretation and modeling of MOKE hysteresis loops.

Supplementary Figure 7 shows a comparison between measured MOKE hysteresis loops and hysteresis loops calculated using the Stoner-Wohlfarth (S-W) model for a ferromagnet with a uniaxial anisotropy. The comparison is made for both magnetic field applied perpendicular to steps (Supplementary Fig. 6a) and along the steps (Supplementary Fig. 6b). In this model, the equilibrium magnetization of the Co is determined by the energy density

$$\frac{E}{V} = -\mu_0 \mathbf{M} \cdot \mathbf{H} + K_\mathrm{U} \sin^2(\theta_\mathrm{Co} - \alpha) \qquad (1)$$

where **M** is the Co magnetization, **H** is the external magnetic field, K_U is the uniaxial anisotropy constant, θ_{Co} is the Co magnetization angle and α is the anisotropy axis angle. The S-W model assumes the Co is a single ferromagnetic domain, which is unrealistic in our 100 μm scale devices but serves as an idealized point for comparison. The parameters used in the model were determined as follows. For the r_1^- state the anisotropy axis direction was chosen to be along the step edge direction and for the r_3^+ state the anisotropy direction was chosen to be 75° from the step edges. For each polarization state the anisotropy energy was determined by the saturation field taken from the experimental data. Additionally, the magnetic field direction was perturbed

by and angle of 2° away from exactly parallel or perpendicular with the surface steps to account for imperfections in the experimental magnetic field alignment. With these choices for our model parameters, a qualitative match is achieved between experiment and calculation indicating that the MOKE and XMCD-PEEM measurements agree.

There are several discrepancies between the S-W model presented and the experimental data. The model systematically overestimates the switching field $\mathbf{H}_c$ to be the point where the Zeeman energy becomes equal to the anisotropy energy. In a sample with magnetic domains magnetic reversal will occur at lower fields due to domain growth if the domain wall depinning energy is smaller than the anisotropy energy. Also, the switching regions of the experimental hysteresis loops show curved features not present in the calculations, especially in the r_1^- state. We believe that these are merely a result of the detailed magnetic reversal process that occurs in magnetic thin films grown on miscut substrates[6] .

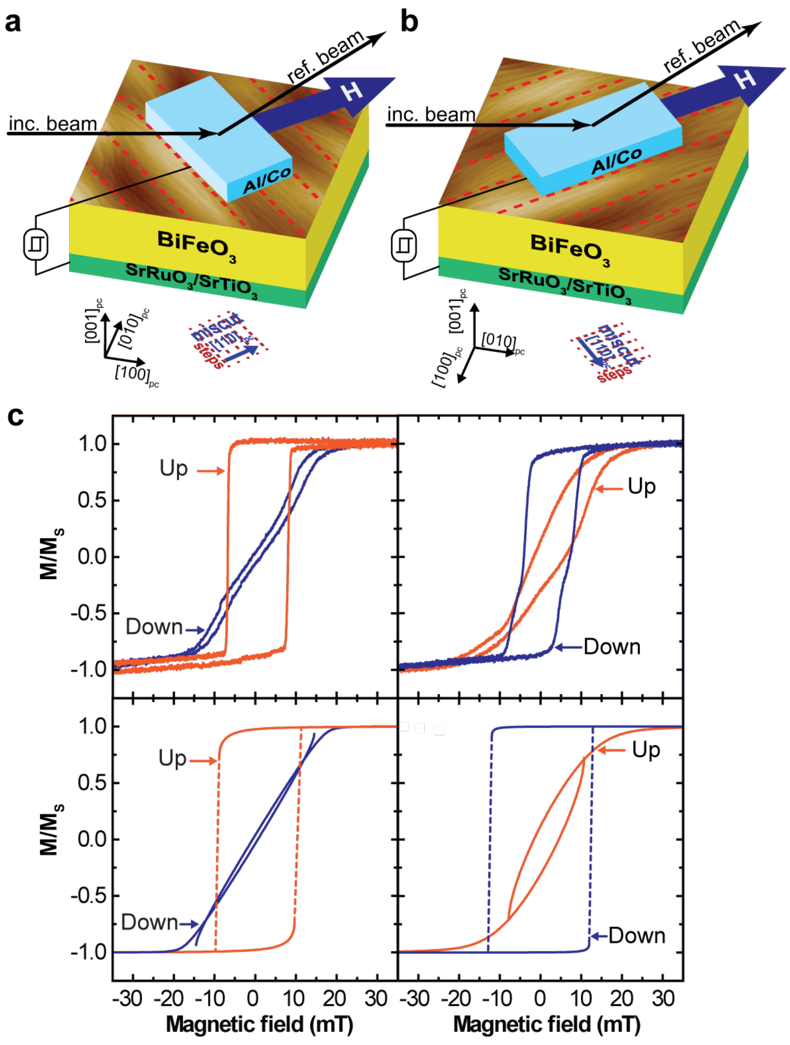

Supplementary Figure 7. Experimental MOKE data compared to a Stoner-Wohlfarth model. Schematics of MOKE experimental configurations for magnetic field applied (**a**) perpendicular to and (**b**) parallel to the surface step edges. (**c**) Comparison between experimental MOKE data (top panels) and theoretical simulation based on S-W model (bottom panels).

Supplementary Note 5. Reproducibility and Robustness to Ferroelectric Cycling

To demonstrate the robustness of the Cobalt anisotropy rotation in our heterostructures, MOKE **M**-**H** loops were measured after 10 and 100 ferroelectric switching cycles (Supplementary Fig. 8a and 8b, respectively) at room temperature. The experimental setup for these MOKE **M**-**H** loops measurement is shown in Supplementary Figure 7a. After 10 cycles the hysteresis loops are akin to those shown in Supplementary Figure 7c which were measured on a different device, and as discussed above demonstrate magnetic anisotropy rotation. After 100 cycles the coercive field has decreased but still the signatures of anisotropy rotation persist. It is likely that oxidation of the Cobalt near the $BiFeO_3$ interface plays a role in the observed device degradation.[7]

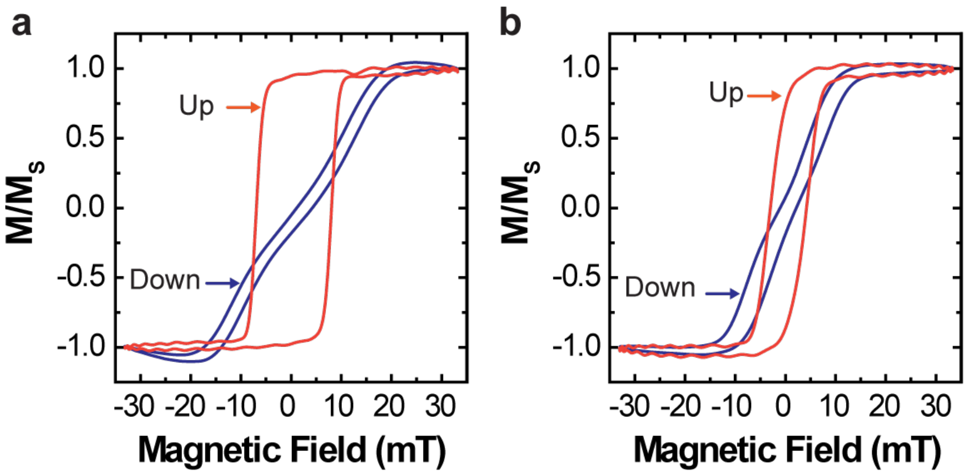

Supplementary Figure 8. Robustness of exchange coupling in Co/$BiFeO_3$ heterostructures. The **M**-**H** loop of Co/$BiFeO_3$ heterostructure after (**a**) 10 ferroelectric polarization switching cycles and (**b**) 100 ferroelectric polarization switching cycles, measured at room temperature.

Supplementary Note 6. XMLD–PEEM imaging of $BiFeO_3$.

PEEM combined with XMLD was performed at several light polarization angles ($\theta_{\mathbf{E}}$) with the XMLD asymmetry defined as

$$I_{\mathrm{XMLD}} = \frac{I_{\mathbf{E}_1} - I_{\mathbf{E}_2}}{I_{\mathbf{E}_1} + I_{\mathbf{E}_2}} \quad (2)$$

where $I_{\mathbf{E}_1}$ and $I_{\mathbf{E}_2}$ are the PEEM image intensities corresponding to specific peaks in the X-ray absorption spectroscopy (XAS) spectrum shown in Supplementary Fig. 9.

For each $BiFeO_3$ ferroelectric polarization state, four sets of PEEM-XMLD images were acquired, scanning θ_{E} from 0° to 90° at sample azimuthal angles of φ_{M} = 0°, 45°, 90° and 135°. According to our calculations[8] the XMLD asymmetry is more positive when the local Néel vector (**L**) is parallel to the electric field of the light (**E**). The crystalline field contribution is neglected since, according to multiplet modeling, it is small[9].

XMLD–PEEM images obtained in the down and up states are shown in Supplementary Fig. 10. The Fe XMLD-PEEM images were acquired on Pt (2 nm)/$BiFeO_3$/$SrTiO_3$ to avoid a reduction of the signal intensity by the Al (3nm)/Co (2nm) layer. We verified that, in the down state, the Al/Co/$BiFeO_3$ film gives the same XMLD results as the bare Pt/$BiFeO_3$ film. The granular background features in both images are due to residual contributions from the detector and surface topography. The down state image reveals a single antiferromagnetic (AF) domain, whereas the up state image exhibits minority AF domains covering <5% of the surface (Supplementary Fig. 10, blue circled areas). The size and contrast of the minority domains are enough to allow detection, but not to determine a spin configuration. Correlating the PEEM images in Supplementary Figure 10 with the PFM images shown in Supplementary Figure 4 reveals that these areas are most likely the 180°–switched (r_1^+) domains. The small contribution to the image intensity from these areas does not change the results of the analysis.

The XMLD images therefore imply the presence of a single ferroelectric domain in the down state, and the presence of a majority domain (r_3^+) covering ~95% of the surface in the up state. Importantly, the presence of these domains shows that the size of the ferroelectric domains is above the spatial resolution of the PEEM.

Supplementary Note 7. Angular dependence of the XMLD intensity.

We can express I_{XMLD} expected from an arbitrary spin distribution $\mathbf{S}_\mathrm{i} = (S_{x_i}\ S_{y_i}\ S_{z_i})$, in laboratory co-ordinates, as a function of the light polarization angle ($\theta_\mathbf{E}$) and the azimuthal rotation angle (φ_M) about the [001] direction as

$$I_{\mathrm{XMLD}}(\theta_\mathbf{E}, \varphi_\mathrm{M}) = d_0 + d_1\ \mathbf{E}_{\theta_\mathbf{E}}\ \mathbf{D}_{\ \varphi_\mathrm{M}}\ \mathbf{E}_{\theta_\mathbf{E}}^\mathbf{T} \tag{3}$$

where d_0 is an offset, d_1 is the XMLD amplitude, $\boldsymbol{E}_{\theta_\mathbf{E}} = [\sin\theta_\mathbf{E} \quad \cos\theta_\mathbf{E} \quad 0]$ represents the polarization vector of the light in laboratory co-ordinates**.** $\mathbf{S}_\mathrm{i}$ can be expressed in matrix form as

$$\mathbf{D} = \frac{1}{N}\sum_i^N \begin{bmatrix} S_{x_i}^2 & S_{x_i}S_{y_i} & S_{x_i}S_{z_i} \\ S_{x_i}S_{y_i} & S_{y_i}^2 & S_{y_i}S_{z_i} \\ S_{x_i}S_{z_i} & S_{y_i}S_{z_i} & S_{z_i}^2 \end{bmatrix} \tag{4}$$

that can be rotated into sample coordinates using

$$\mathbf{D}_{\varphi_\mathrm{M}} = \mathbf{R}_{\varphi_\mathrm{M}}\mathbf{D}\ \mathbf{R}_{\varphi_\mathrm{M}}^\mathbf{T} \tag{5}$$

To simulate the XMLD data in Fig. 3(a) and (b) another rotation of **D** is made beforehand, resulting in the fitting parameters α and β, representing a rotation of $\mathbf{S}_\mathrm{i}$ about $[110]_{\mathrm{pc}}$ and $[\bar{1}\bar{1}0]_{\mathrm{pc}}$**.** respectively.

The fitting of the XMLD data shown in Fig. 3a used d_1 as a fitting parameter, with $\mathbf{S}_\mathrm{i}$ constrained by α and β to be a spin cycloid in the film plane as determined by the ND results. The fitting of the XMLD data shown in Fig. 3b followed the same procedure with an additional out-of-plane component allowed for $\mathbf{S}_\mathrm{i}$ (a rotation of the spin cycloid plane through α or β could not fit the XMLD data for the up state).

To get a better insight into the changes to the Fe spin cycloid after poling from the down state to the up state, a second procedure was used. For the second approach, **D** was constrained to be diagonal with eigenvalues (*a b c*). The eigenvalues (*a b c*) and the rotation angles (α and β) were used as fitting parameters. In this way, an out-of-plane collinear AF structure along the *z*-axis can then be represented by the eigenvalues ($a = 0$, $b = 0$, $c = 1$), while a spin cycloid in the *x-y* plane can be represented by the eigenvalues ($a = 1$, $b = 1$, $c = 0$).

The key point here is that this approach allows insight into whether the polarization switching of the $BiFeO_3$ rotates or distorts the Fe spin cycloid, i.e. if the pure cycloid order is lost

at the interface in the up state or whether a new interface component must be considered to account for the out of plane distortion of $\mathbf{S}_i$ in a way that determining $\mathbf{S}_i$ in matrix form by fitting the XMLD data does not.

The fitting of the XMLD data shown in Fig. 3(a) for the down state constrained a to 1, to avoid cross-correlation with d_1, and c to 0. The fitting then determined d_1, b, α and β. The fitting results for the down are shown in Supplementary Table 2.

The best fit was found for b=1±0.1 (Fig. 3(a)), indicating that the spin distribution can be considered as a spin cycloid (*i.e.* a spin distribution with axial symmetry) in the film plane. The spin cycloid is found to be rotated by $\alpha = -8°$ (namely towards the $[00\bar{1}]_{pc}$ direction) with $\beta = 0$. This solution is in excellent agreement with the ND results for the down state. For the up state, d_1 was constrained to the value determined for the down state, with $a = b = 1$. The fitting then determined c, α and β. The fitting results are shown in Supplementary Table 2 and indicate the appearance of an additional out-of-plane component given by $c = 0.5 \pm 0.06$ (Fig. 3b). This shows that the spin distribution of the $BiFeO_3$ in the interface region changes after switching from the down to the up state. A simple rotation of the spin cycloid can be ruled out since such a rotation would leave the eigenvalues unchanged. The implication is that the up state out-of-plane component represents either an interface induced collinear AF moment or an additional interface spin-cycloid with an out-of-plane component. However, for a thin film with strain, an out-of-plane spin cycloid is not likely. We therefore modelled the up state as the sum of the bulk cycloid, as measured by ND, and a surface collinear AF with **L** perpendicular to **P** with the relative contributions left as a fitting parameter. The best agreement was obtained for a bulk cycloid with a ~1 nm thick interface region with **L** oriented along $[112]_{pc}$. The exchange coupling to the Co would then presumably occur via uncompensated interfacial Fe moments along **L**.

Simulations of I_{XMLD} with θ_E assuming the bulk spin cycloid orientations for the down and up states are shown in Fig. 3c and 3d and Supplementary Fig. 11. The simulations do not fit the experimental XMLD data. The spin cycloid plane for the thin film in the down or up state therefore does not have any of the 3 allowed orientations for bulk single crystals, in agreement with the ND results.

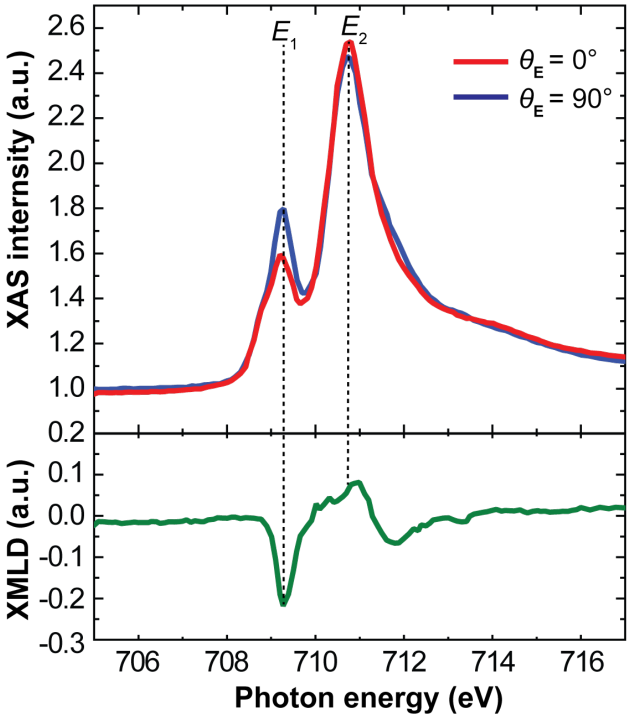

Supplementary Figure 9. XAS spectra. XAS spectra from Pt(2 nm)/BiFeO3 over the Fe L_3 edge measured with PEEM for $\boldsymbol{\theta}_{\mathbf{E}}$=0 (red line) and $\boldsymbol{\theta}_{\mathbf{E}}$=90º (blue line) showing the two energies E_1 and E_2. The difference between the two spectra is shown as the XMLD (green line).

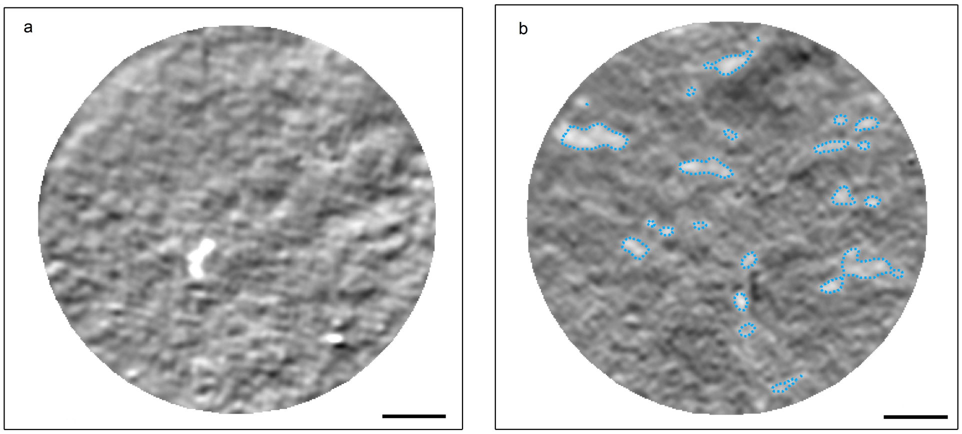

Supplementary Figure 10. XMLD-PEEM images showing AF domains. XMLD-PEEM image of the Pt (2 nm)/$BiFeO_3$ in the (**a**) down state and (**b**) up state for $\boldsymbol{\varphi}_{\mathbf{M}}$ = 0 and averaged over 30º ≤ $\boldsymbol{\theta}_{\mathbf{E}}$ ≤ 50º showing the presence of minority AF domains (blue-circled white areas). The white area in (a) is a defect. Scale bars, 2 μm.

Parameters	down state	up state
d_1	0.064 ± 0.01	0.064
α	$-8^{o} \pm 3^{o}$	$-8\,^{o} \pm 3^{o}$
β	0 ± 2°	0 ± 2°
**D**	$\begin{bmatrix} 1 & 0 & 0 \\ 0 & 1 \pm 0.1 & 0 \\ 0 & 0 & 0 \end{bmatrix}$	$\begin{bmatrix} 1 & 0 & 0 \\ 0 & 1 & 0 \\ 0 & 0 & 0.5 \pm 0.06 \end{bmatrix}$

Supplementary Table 2. **XMLD fitting parameters.** XMLD amplitude (d_1), main axis orientation (α, β) and **D** obtained from fitting the XMLD angle dependent data. Values without error bars were constrained.

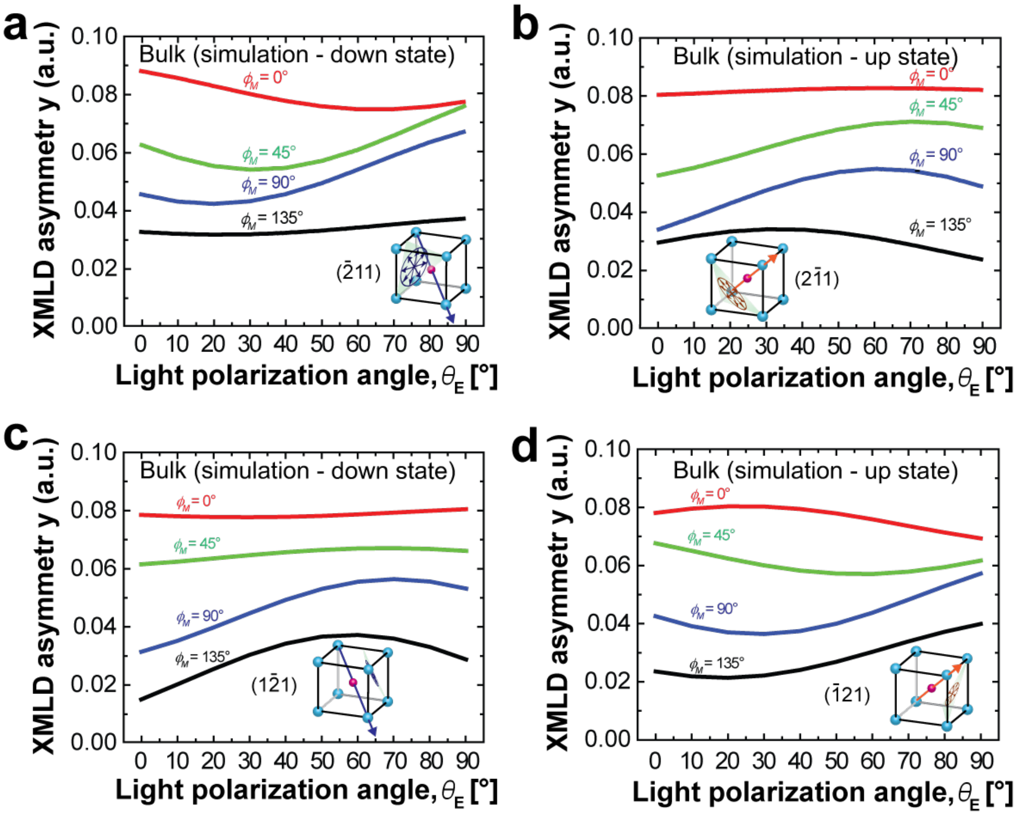

Supplementary Figure 11. XMLD simulations. Additional simulations of the bulk spin cycloid planes in the (**a,c**) down state and (**b,d**) up state. The bulk spin cycloid planes in **a**, **b**, **c** and **d** are $(\bar{2}11)$, $(2\bar{1}1)$, $(1\bar{2}1)$ and $(\bar{1}21)$, respectively.

Supplementary Note 8. XMCD–PEEM determination of the local Co moment rotation.

By making the difference of two Co magnetization vector maps (Fig. 4) taken across the switching of the $BiFeO_3$ we can extract the local rotation for each magnetic domain. The distribution of the rotation angle is broad and peaks at ±70° (Supplementary Fig. 12c). The result of the rotation is well described by the polar plot of the XMCD-PEEM vector map of the Co film (Fig. 4): in the down state the lobes of the distribution are aligned perpendicular to $[110]_{pc}$ (Fig. 4d and 4f), while in the up state the domains are rotated ~70° - 90° towards the $[110]_{pc}$ direction (Fig. 4e).

These results can be explained in terms of a competition between two magnetic anisotropies: a uniaxial anisotropy perpendicular to $[110]_{pc}$ arising from the miscut, and an anisotropy arising from the creation of the interface collinear AF state in the up state. The estimated value of the latter energy term obtained by fitting the MOKE **M**-**H** hysteresis loops (using a macro-spin model) leads to a predicted rotation angle of ~75° in good agreement with the XMCD-PEEM results.

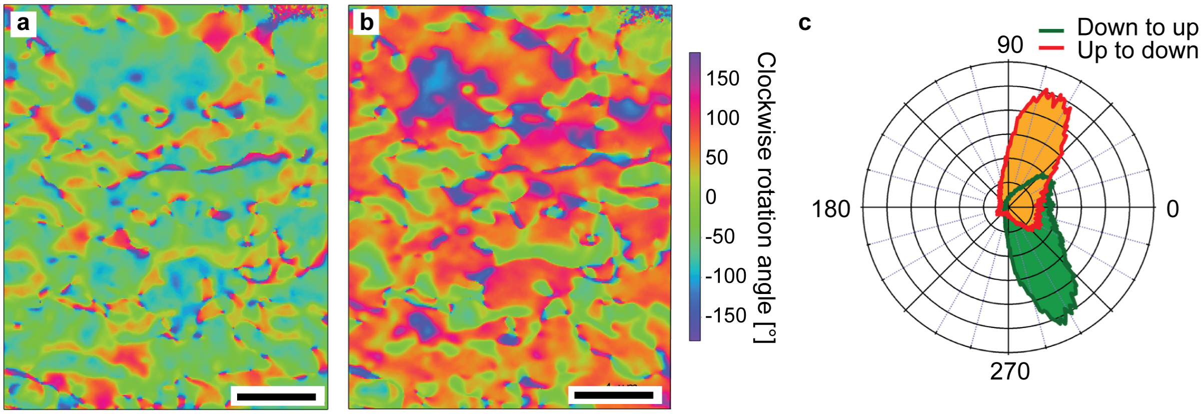

Supplementary Figure 12. Co magnetic rotation map upon ferroelectric polarization. XMCD-PEEM difference maps of the local Co spin rotation following polarization switching from (**a**) down–up and (**b**) up–down. (**c**) Distribution of the local spin rotation after poling down–up (green) and up–down (orange). Scale bars, 4 μm.

Supplementary References

1 Jang, H. W. et al. Domain Engineering for Enhanced Ferroelectric Properties of Epitaxial (001) $BiFeO_3$ Thin Films. *Adv. Mater.* **21,** 817-823, (2009).

2 Kim, T. H. et al. Step bunching-induced vertical lattice mismatch and crystallographic tilt in vicinal $BiFeO_3$ (001) films. *Appl. Phys. lett.* **98,** 022904, (2011).

3 Johnson, R. D. et al. X-Ray Imaging and Multiferroic Coupling of Cycloidal Magnetic Domains in Ferroelectric Monodomain $BiFeO_3$. *Phys. Rev. Lett.* **110,** 217206, (2013).

4 Bucci, J. D., Robertson, B. K. & James, W. J. The precision determination of the lattice parameters and the coefficients of thermal expansion of $BiFeO_3$. *J. Appl. Crystallogr.* **5,** 187-191, (1972).

5 Sosnowska, I., Peterlin-Neumaier, T. & Steichele, E. Spiral magnetic ordering in bismuth ferrite. *J. Phys. C: Solid State Phys.* **15,** 4835-4846, (1982).

6 Hyman, R. A., Zangwill, A. & Stiles, M. D. Magnetic reversal on vicinal surfaces. *Phys. Rev. B* **58,** 9276-9286, (1998).

7 Couet, S. et al. Electric Field-Induced Oxidation of Ferromagnetic/Ferroelectric Interfaces. *Advanced Functional Materials* **24,** 71-76, (2014).

8 Czekaj, S., Nolting, F., Heyderman, L. J., Willmott, P. R. & van der Laan, G. Sign dependence of the x-ray magnetic linear dichroism on the antiferromagnetic spin axis in $LaFeO_3$ thin films. *Phys. Rev. B* **73,** 020401(R), (2006).

9 Ko, K. T. et al. Concurrent transition of ferroelectric and magnetic ordering near room temperature. *Nature Commun.* **2,** 1567, (2011).
